# Breastfeeding Journeys: Comparing Mothers’ Experiences with Autistic and Neurotypical Infants

**DOI:** 10.1007/s10803-024-06669-9

**Published:** 2024-12-19

**Authors:** Amy A. Campbell, Julie Barroso, Mulubrhan Mogos, Amy Weitlauf, Sharon M. Karp

**Affiliations:** 1 Vanderbilt University School of Nursing, Nashville, TN USA; 2 Vanderbilt University Medical Center, Nashville, TN USA

**Keywords:** Autism spectrum disorder, Breastfeeding, Feeding difficulty, Infant feeding

## Abstract

Breastfeeding is a complex task that requires proficiency at several key developmental skills to feed successfully. It is unclear how Autism Spectrum Disorder (ASD) affects an infant’s breastfeeding experience and conflicting reports exist on shortened breastfeeding duration in infants later diagnosed with ASD. The purpose of this study was to describe the experiences of mothers breastfeeding both their autistic and neurotypical children to better understand the differences in their breastfeeding experiences and maternal and infant factors that contributed to breastfeeding cessation in their autistic child. Twenty-four mothers of an autistic child who had feeding difficulties in the first 12 months of life, initiated breastfeeding, and also had a neurotypical child participated in semi-structured interviews regarding breastfeeding initiation, cessation, and challenges encountered during breastfeeding. Analysis revealed four major themes: (1) the struggle with latch, including infant behavior that hindered latching; (2) breastfeeding challenges, including problematic breastfeeding behavior by the infant; (3) cessation of breastfeeding, including physical symptoms of the infant that lead to breastfeeding cessation; and (4) breastfeeding the neurotypical sibling, including mother’s detection of different breastfeeding behaviors in neurotypical and autistic child. Breastfeeding behaviors described by mothers may indicate early signs of autism including early sensory sensitivity, lack of regulation, repetitive behaviors, and impaired social behaviors. Further research is needed to discern if these breastfeeding behaviors can be used to help identify early signs of autism and employed as additional surveillance for neurodevelopmental concerns at a young age.

## Introduction

Autism Spectrum Disorder (ASD), impacting 1 in 36 children in the United States (U.S), is a neurodevelopmental condition characterized by persistent deficits in social communication and interaction, as well as repetitive and restricted patterns of behavior and interests (American Psychiatric Association [APA], 2022). Autism Spectrum Disorder can be reliably diagnosed by a specialist by 2 years of age, yet in the U.S., the median age of diagnosis is 46–56 months (CDC, 2023). This delay in diagnosis is critical because early therapy initiation can lead to better outcomes (Sandbank et al., 2023). Although diagnosis usually occurs after age 2 years, many parents report noticing signs of ASD much earlier, suggesting that screening and diagnosis may be enhanced by asking parents questions about infant behavior (Dawson et al., 2023). Therefore, there is an urgent need for the field to identify very early behavioral indicators of neurodevelopmental deficits to enable early therapy initiation and maximize the potential for positive developmental trajectories.

One early indicator of a need to monitor a child for neurodevelopmental differences like autism may be behavior during breastfeeding. Breastfeeding is a complex developmental task that uses motor and cognitive skills by an infant and reliance on the mother to provide milk (Manella et al., 2020). Suckling is the primary feeding milestone and, along with swallowing and breathing, the nervous system provides the infant a coordinated way to feed without stopping to take breaths (Dodrill, 2021). In addition to these neuromotor tasks for effective breastfeeding, infants must also participate in the breastfeeding process by communicating hunger and satiation, regulating intake, and responding to maternal cues where behavior modification during feeding may be needed (Manella et al., 2020). An interruption in any of these key developmental skills (neuromotor, cognitive, and communication) can make successful breastfeeding difficult. Because the core features of ASD include social communication deficits and restricted and repetitive patterns of behavior that include hyper- or hypo-reactivity to sensory stimuli (APA, 2022), determining breastfeeding behaviors that may exhibit these core features may be beneficial in identifying earlier autistic traits. How ASD affects the breastfeeding experience of children and their mothers remains unclear, and the potential of breastfeeding experiences as an early marker of the need for developmental monitoring for ASD has not been thoroughly explored.

The American Academy of Pediatrics (AAP, 2022) recommends that infants be exclusively breastfed for the first 6 months of life. However, there have been reports of autistic children breastfeeding for a shorter duration than neurotypical children (Lemcke et al., 2018; Soke et al., 2019). In a meta-analysis done by Tseng et al. (2019), it was found that significantly fewer autistic children were breastfed beyond six months of age when compared to neurotypical children. Using the National Survey of Children’s Health data in the United States, Zhan et al. (2023) compared trends in rates of exclusive breastfeeding during the first 6 months of life in autistic and non-autistic children. In this study, the rate of exclusive breastfeeding during the first six months of life decreased from 12.0% in 2016 to 4.2% in 2020 in autistic children, whereas in non-autistic children, the rate of exclusive breastfeeding increased from 6.4% in 2016 to 9.8% in 2020. On the contrary, there have been other studies that have not found significant differences in breastfeeding duration or practices in autistic children (Burd et al., 1988; Emond et al., 2010; Husk & Keim, 2015). The Avon Longitudinal Study of Parents and Children (ALSPAC) found no significant difference in breastfeeding rates at 6 months of age between autistic and non-autistic children (Emond et al., 2010) and Husk and Keim (2015) did not find a significant difference in duration of exclusive breastfeeding and subsequent autism diagnosis.

Because only quantitative data has been presented in these studies on breastfeeding duration in autistic and non-autistic children, it is difficult to determine what additional causes may have contributed to earlier breastfeeding cessation in autistic children as reported in a portion of these studies. There is a paucity of qualitative data as to the reasons why mothers of an autistic child quit breastfeeding that would help explain in these studies why the breastfeeding duration was significantly decreased in autistic children. The purpose of this study was to describe the experiences of mothers breastfeeding their infant later diagnosed with ASD and their neurotypical child to better understand maternal and infant factors that contributed to breastfeeding cessation and the differences in breastfeeding experiences in their autistic child and neurotypical child.

## Conceptual Framework

**Fig. 1:**
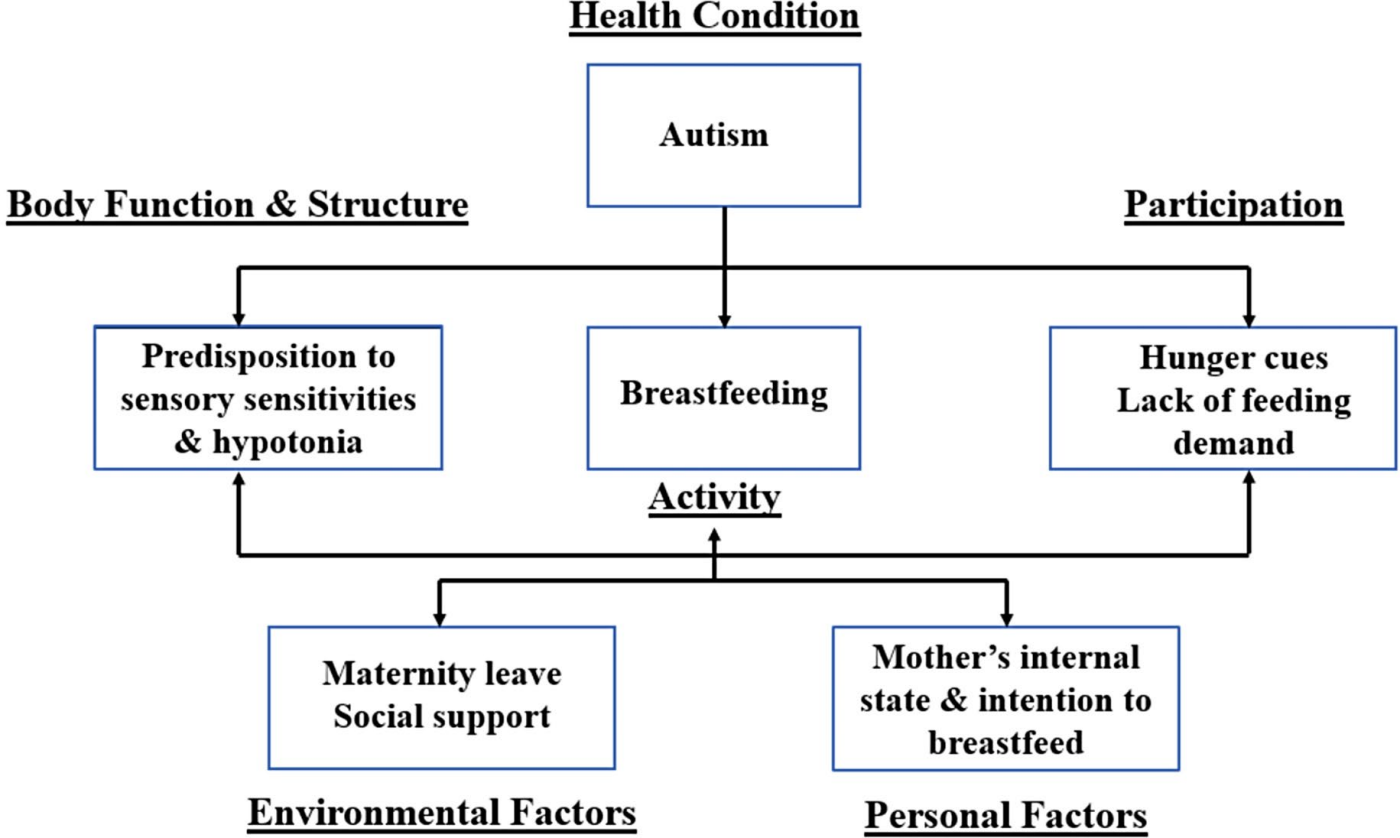
Adapted International Classification of Functioning, Disability & Health (ICF) Framework

This study was guided by the International Classification of Functioning, Disability, and Health (ICF) framework (see Fig. 1) (USDHHS, 2001). The ICF is a biopsychosocial model and its concepts interact to display an ever-changing relationship between health conditions, personal factors, and environmental factors to represent the multi-faceted nature of feeding (Morris et al., 2017). The ICF’s focus on Functioning and Disability are displayed by several concepts within the model to portray body function and impairment or restrictions in activity or participation in daily life tasks. The ICF framework has been adapted for this study to include breastfeeding as the focused activity with Autism being the focused health condition (USDHHS, 2001). Feeding is multifaceted; therefore, when identifying infant feeding characteristics in ASD many factors may contribute to the feeding difficulty including anatomic, behavioral, contextual, developmental, and medical factors (Morris et al., 2017).

In using the ICF’s major concepts to investigate breastfeeding behaviors in infants later diagnosed with ASD, the Body Function and Structure domain could include conditions that autistic children are more predisposed to, such as sensory sensitivities and hypotonia (Gabis et al., 2021; Raj et al., 2023), which both affect feeding functioning such as sucking. The Participation domain could include feeding refusal, hunger cues, and dysregulation of breastfeeding demand, all of which can contribute to a decrease in the mother’s breast milk supply and limit the capacity to breastfeed (Daly & Hartmann, 1995). Personal factors include the mother’s attitude and intention to breastfeed and postpartum depression/anxiety that may influence breastfeeding practices. Environmental factors that may contribute to breastfeeding difficulty include length of maternity leave, workplace environment to support pumping while away from the infant, and social support of the mother to help with breastfeeding difficulty (McKinley & Hyde, 2004). The concepts of Participation, Body Function and Structure, Environmental Factors, and Personal Factors included in the ICF allow us to examine feeding behaviors reported in infants later diagnosed with ASD from many different contexts and their influence on the mother’s perceptions of their child’s feeding characteristics.

## Methods

This study used a qualitative descriptive design with semi-structured interviews to obtain information from mothers on the breastfeeding characteristics and challenges they experienced with their infant later diagnosed with ASD. The outcome of a qualitative descriptive study is a straightforward, descriptive summary of the data obtained and presented in a way that is most fitting for that data (Sandelowski, 2000).

Mothers were recruited from national autism organization websites, social media sites, ResearchMatch, local pediatric primary care office flyers, and a university affiliated autism clinic research listserv. Inclusion criteria for the study were English-speaking mothers of children with an ASD diagnosis aged 2–12 years old who had a history of feeding difficulty in infancy (0–12 months of age), who did initiate breastfeeding, and who have at least one additional neurotypical child that breastfed as well. Exclusion criteria included mothers of children born before 37 weeks gestation and those with known chromosomal, anatomic, or additional medical comorbidities that may affect feeding behavior in infancy. These excluded medical comorbidities included craniofacial malformations (e.g., cleft lip / palate), diagnosed chromosomal mutation (e.g., Down Syndrome), congenital heart disease, and cerebral palsy due to these conditions’ potential to immediately impact feeding after birth (Reilly et al., 2006).

Study procedures included a short verbal demographic survey and a semi-structured qualitative interview. The interviews were conducted on Zoom with both audio and video enabled, lasting between 30 and 60 min. In one case, internet connection was lost, and the remainder of the interview was completed over the telephone. The principal investigator (AC) conducted each interview. Informed consent was obtained from each participant and the study was approved by the Vanderbilt University Institutional Review Board.

The semi-structured interview questions were created using the guidance of the study’s conceptual framework, the ICF, and included questions and prompts about sucking, swallowing, latch, frequency of feedings, rationale and timing of breastfeeding cessation, accessory use to aid in breastfeeding, breastfeeding neurotypical sibling, breastfeeding support, and the emotions experienced with breastfeeding. The questions encompassed the time period of the initiation of breastfeeding right after delivery to the end of their breastfeeding experience when cessation occurred.

All interview recordings were transcribed verbatim and the transcripts checked against the recordings to ensure accuracy. Interview recordings, transcripts, and codebooks were all secured in HIPAA-compliant data storage software. Coding of the transcripts was done using a qualitative descriptive technique which ultimately provides a rich description of the data and provides a more detailed description than what quantitative data can provide (Sandelowski, 2000).

The first 10 transcripts were reviewed and coded by two reviewers (AC and JB) and the remaining transcripts were coded by a single reviewer (AC). Codes that encompassed the mothers’ experiences of breastfeeding both their autistic as well as their neurotypical child were extracted from all of the codes of the overall study. These codes were then placed into several categories that encompassed varying topics related to breastfeeding: challenging breastfeeding behaviors, latching issues, mothers’ experiences breastfeeding their neurotypical children, and their reasons for stopping breastfeeding. Data reduction then occurred where similar codes were collapsed into one code and codes that may not be directly related to breastfeeding were removed. During the data reduction process, quotes from the interviews were checked to ensure crucial data was not being eliminated. Following data reduction, the four categories originally created became the overarching themes. The codes within these themes were then delineated to create subthemes. Peer debriefing, a complete audit trail, and being reflexive by writing reflections after each interview to enhance trustworthiness that the results accurately represent the participants’ experiences were all done to enhance rigor of the research.

## Results

### Participants

Participants included 24 mothers of infants later diagnosed with autism (hereafter referred to as “index infant”) who had feeding difficulty during the first year of life. All of the participants reported that their child received an ASD diagnosis from either a psychologist, neurologist, developmental pediatrician, or psychiatrist. The average age of the mothers was 37.8 years and the average age of the autistic child was 7.5 years. The average age at the time of autism diagnosis was 46.3 months. The majority (87.5%) of the mothers had a male autistic child and 83.3% of the mothers were White. The mothers resided in 12 different states and 66.7% of the mothers had a bachelor’s degree or higher. The majority of the autistic children were not the first born child (62.5%). Table 1 provides a summary of demographic characteristics of the participants.

**Table 1:** Summary of demographic characteristics of participants (*n* = 24)

Variable	Mean (SD)
**Mother’s Age (years)**	37.8 (5.7)
**Present Age of Autistic Child (years)**	7.5 (2.6)
**Present Age of Neurotypical Sibling (years)**	9.4 (4.6)
**Age of Child When Diagnosed with ASD (months)**	46.3 (27.3)
	**N(%)**
**Sex of Child**	
Male	21 (87.5)
Female	3 (12.5)
**Education Level of Mother**	
High school diploma	3 (12.5)
Associate’s degree	5 (20.8)
Bachelor’s degree	6 (25.0)
Master’s degree	9 (37.5)
Doctoral degree	1 (4.2)
**Ethnicity of Mother**	
White	20 (83.3)
Black	1 (4.2)
Asian	2 (8.3)
Hispanic	1 (4.2)
**Birth Position of Autistic Child**	
First born	9 (37.5)
Second born	10 (41.7)
Third born	5 (20.8)

### Qualitative Results

**Fig. 2:**
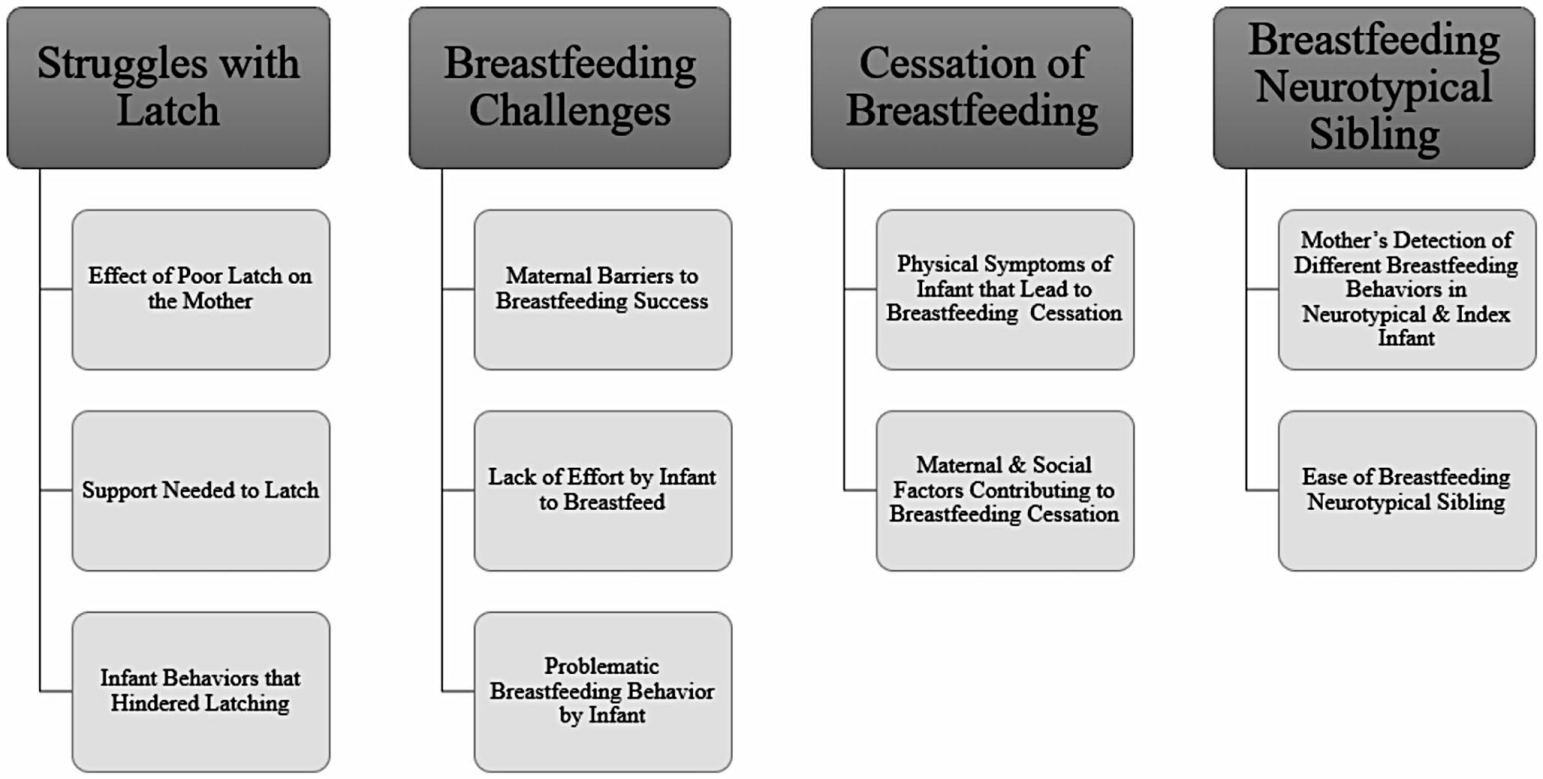
Themes and subthemes from qualitative analysis

Four themes were developed from the coding process: struggles with latch, breastfeeding challenges, breastfeeding the neurotypical sibling, and cessation of breastfeeding with subthemes that emerged for each theme. Themes and subthemes are displayed in Fig. 2. Count responses for concerning breastfeeding behaviors in the autistic children are included in Table 2.

**Table 2:** Participant count responses for concerning breastfeeding behaviors in autistic child

Concerning Breastfeeding Behavior in Autistic Child	Number of Participants Reporting Concern (*n* = 24 participants)
Difficulty latching	14
Lack of effort / stamina by infant to breastfeed	11
Need for nipple shield (after 2 weeks of age)	10
Short amount of time breastfeeding	7
Infant very particular of position to breastfeed	6
Painful latch	5
Uncoordinated latch and suck	5
Delayed latching success (2 weeks or longer)	4
Choking / Gagging while breastfeeding	4
Lack of cues by infant to indicate satiety / hunger	3
Breastfeeding very slowly	1

### Struggles with Latch

Three subthemes supported this overarching theme and were: effect of poor latch on the mother, support needed to latch, and infant behaviors that hindered latching.

#### Effect of Poor Latch on the Mother

Five of the mothers reported a painful latch that continued well into infancy, and for some, the pain persisted for months. One of the infants did have a tongue and lip tie revision while the other infants were either not assessed or not found to have anatomic abnormalities that could contribute to a painful latch. One mother described the pain as “I didn’t have a single breastfeed that didn’t feel like a cheese grater ripping my nipples off. Not a single time.”

Multiple mothers had delayed latching success that took two or more weeks to achieve a successful latch with their index infant or they successfully latched in the hospital but were unsuccessful latching after discharge. These latching issues caused mothers to pump and bottle feed expressed breastmilk which the mothers felt hindered their success in breastfeeding as the infant got used to using a bottle. One mother spoke of her experience after hospital discharge of her difficulties getting her index infant to latch:

So I guess objectively, what I should say is we did get a successful latch for the very first time right after he was born. It was right to the breast and he fed for a little while. And we felt great at first, at that first moment…. So then moving on to going home and everything, I found it was just so difficult to get him to latch. And of course it being my first child, I assumed it was all of my fault.

#### Support Needed to Latch

Ten mothers reported the need to use a nipple shield while two other mothers who had latching difficulty and pain with latching never received a recommendation to try a nipple shield to assist with latching. One mother spoke of her experience using a nipple shield for an entire year so that she could successfully breastfeed her index infant:

It was just impossible for him to latch. What finally ended up working was I had to use, all the way through his first year, was the nipple shield. I was very sleep-deprived that year, so it was just like going through the motions and the constant cleaning of the nipple shield. I had multiple, but at the same time, you need to sanitize it and everything. When you’re done feeding, you’re not done. You need to sanitize, you need to wash.

#### Infant Behaviors that Hindered Latching

Fourteen of the 24 mothers reported difficulty getting their index infant to latch because of infant behaviors that impeded the latching process. Multiple mothers experienced a short-lived latch and a lack of effort and stamina by the infant to stay on the breast despite an absence of medical conditions that would contribute to decreased stamina with breastfeeding. One mother recalls, “He couldn’t latch. We had a lot of problems with latching and even if he would latch, it wouldn’t last very long. So, he really struggled with figuring out the concept of latching and also putting forth enough effort to get enough milk to sustain himself.”

Mothers noticed other characteristics of their index infant’s latching that made successful latching difficult such as an unsteadiness with the latch, screaming every time latching was attempted or right after infant latched, infant’s preference to be in a very particular position on the breast to latch successfully, difficulty understanding how to latch, and an uncoordinated latch. One mother tells of how the uncoordinated latch of her index infant transitioned to uncoordinated eating even as he grew older:

With him being my third, I had breastfed before, so I wasn’t completely inexperienced, so let’s start with that. He couldn’t latch. Remained the problem forever. He was really uncoordinated is what they called it, I guess. So yeah, the latch seemed to be the problem, but move forward, still today he still has problems eating and it’s something with chewing, so it still almost is the same, even though he’s much older now.

### Breastfeeding Challenges

In addition to latching issues, there were several reasons identified as to what caused challenges in successfully breastfeeding their index infant: maternal barriers to breastfeeding success, lack of effort by infant to breastfeed, and problematic breastfeeding behavior by infant.

#### Maternal Barriers to Breastfeeding Success

The majority of maternal barriers the participants identified in successfully breastfeeding their index infant were physical barriers. Five of the mothers stated they did not have an adequate milk supply and one mother reported that she had inverted nipples which caused latching and breastfeeding to be more difficult. Mastitis also interfered with breastfeeding success with their index infant and one mother questioned “Because of the breast infection, does my milk taste bad?” as her index infant would turn his head away and refuse to nurse.

In addition to physical challenges that mothers experienced in breastfeeding successfully, there were mental challenges that mothers faced as well. One mother reported experiencing generalized anxiety and that her index infant would not breastfeed when he sensed the mother’s anxiety. Another mother who had recently received an autism diagnosis herself and also had decreased milk supply struggled with pumping that she needed to do to increase her milk supply. She recalls, “And that was one of my biggest struggles remembering to pump. Pumping in general, I mean, it makes me cringe. It was torture on my sensory system, but I knew that I had to do that because I wanted to be a good mom.”

#### Lack of Effort by Infant to Breastfeed

Eleven mothers reported that breastfeeding their index infant was challenging due to the infant lacking effort or the desire to breastfeed. This lack of effort was displayed in various behaviors including losing interest in sucking and unlatching, short feeding times, being on and off the breast constantly, and feeding very slowly. One mother recalls the lack of interest her infant had while breastfeeding:

“He would lose interest quickly, even though he was hungry. I know that sounds weird, but that pretty much what would happen, and I couldn’t tell if it was because he wasn’t getting what he needed or if he was actually… I think seriously, he was just losing interest not because he wasn’t hungry or he couldn’t figure it out, but he just would lose interest and stare off in the distance.”

Mothers also struggled with keeping their infant awake long enough on the breast to get a full feeding in. This contributed to less transfer of milk from mother and infant not receiving a full feeding, which caused the mother to have to pump and bottle feed the remainder of the milk for that feeding. One mother describes her son’s prolonged sleepiness on the breast:

But he wanted me as a pacifier, but he would just start eating and within seconds fall asleep. I know that’s normal for the first month or so, but it was like the entire year.

#### Problematic Breastfeeding Behavior by Infant

Eight of the mothers reported that certain oral motor behaviors contributed to difficulties breastfeeding their index infant. These behaviors included choking and gagging while breastfeeding, swallowing difficulties and keeping up with mother’s milk letdown, uncoordinated suck, and exhibiting behaviors of oral sensitivity (e.g., sensitivity to touch, texture, and taste of the tongue, lips, palate, and face [Dodrill et al., 2004]). None of these infants were born with any apparent oral motor defect or chromosomal disorder that would hinder breastfeeding initially at birth. However, one mother whose daughter was later diagnosed with dysphagia at a few months of age recalls very early on the difficulties she had breastfeeding:

So she was a couple of days old, and I remember I was trying to nurse her and it was like she was choking, and she legitimately seemed like she was choking, and she was just a few days old, and I thought, that’s so weird that she’s choking, she’s nursing, so she’s in control of the pace.

In addition to challenging oral motor behaviors, thirteen mothers recalled social emotional behaviors by the infants that made breastfeeding more challenging. The majority of these mothers experienced either a lack of social cues from their infants to indicate hunger or satiety or felt that their infant was constantly hungry despite adequate milk intake. One mother describes her son’s insatiable appetite when breastfeeding as an infant as “He just wanted to eat and eat and eat and eat. I’ve had two other kids and they were not like this. He just, every hour, all day, all night, was starving. And I am an over-producer. It was not for lack of milk.”

Another mother describes her experience with her daughter while breastfeeding about the challenge of having a lack of typical hunger cues for infants such as rooting or bringing her hands to her mouth that led to every 2 h feedings that continued for the first three years of her life:

“So whenever she felt hungry she would scream really, really loud. So it wasn’t even just showing any signs that she was hungry, she would just start screaming. So it was really hard to actually figure out when she was hungry, so we actually had to follow a schedule on her feeding.”

Six of the mothers also noted that their infant was very particular about the positioning they were in while breastfeeding and that the position had to be the same each time in order for breastfeeding to be successful. For some mothers, the positioning was very difficult on their bodies and made it challenging to breastfeed anywhere outside the home. One mother recalls her daughter’s particular position while breastfeeding:

So with her, I had to have her sitting up when I was breastfeeding with her. So a lot of the times I’ve actually had her in a harness and have her breastfeeding, because she did not like to lay on my arm or on a Boppy pillow or anything when she was feeding. She had to be sitting up, and it had to be, I don’t know how it was, but it had to be where it was perfect and if she moved, she still had it right there. And then she would get very, very fussy if it did slip out, she would start pinching and scratching a lot.

### Cessation of Breastfeeding

During the interviews, mothers were asked about how long they breastfed their index infant as well as the reasons behind weaning. The range of breastfeeding duration for the group of participants was 2 weeks to 2.5 years. Mothers identified physical, social, and emotional factors that contributed to breastfeeding cessation.

#### Physical Symptoms of Infant that Lead to Breastfeeding Cessation

Nine mothers attributed breastfeeding cessation to physical symptoms in their index infant that indicated breastfeeding was no longer the best nutritional option for them. Slow growth, persistent fussiness with breastfeeding, gastrointestinal symptoms such as colic and constipation, the need to thicken liquids to feed safely, persistent pumping due to poor latch, and lack of satiation were all causes for the mothers to stop breastfeeding. One mother who struggled with her daughter’s regulation of breastmilk intake explains her cessation experience:

So with my daughter, when she started breastfeeding everything went well, but there was days where she just did not want to eat, and then there was days where she would constantly overfill where she was projectile vomiting…and the feeding was hard, so I stopped breastfeeding at three and a half months.

#### Maternal and Social Factors Contributing to Breastfeeding Cessation

Eight of the mothers attributed weaning their infants because of external factors that no longer made breastfeeding feasible such as starting school, terminal illness in a family member, and returning to work. Maternal barriers to continue breastfeeding their index infant included decreased milk supply, mother’s anatomy that impeded breastfeeding such as inverted nipples, and pregnancy with a subsequent child. One mother who was not able to produce enough milk to feed her index infant and decided to wean him at 2 weeks of age emotionally retold her experience of weaning her son:

I just felt, no matter how I tried to express milk, whether it was pumping or feeding for him… I think part of that was a contributing factor of, I just didn’t produce. I just switched to formula. At that point I was like, ‘I’m driving myself nuts.’ It was definitely much more of a grieving process though too, and a lot more of why didn’t this work? What did I do wrong type of thing.

During the interviews, the emotions tied to weaning were still evident with the mothers as they recalled even years later the factors that caused them to wean their infants. One mother reflected even years later that, “And yes, there is a lot of shame and sadness surrounding weaning a lot, kind of makes me emotional to talk about it.”

### Breastfeeding Neurotypical Sibling

All of the participants breastfed both their index infant and their neurotypical child. During the interviews, mothers were asked questions to compare their breastfeeding experiences between their two children. Mothers were able to identify differences in the breastfeeding behaviors of the index infant and the neurotypical infant and also the ease with which their neurotypical child breastfed. Table 3 displays the feeding challenges mothers experienced breastfeeding their autistic child compared to their neurotypical child.

**Table 3:** Differences mothers reported in breastfeeding their autistic vs. neurotypical child

Autistic Child	Neurotypical Child
Lack of hunger cues (e.g., crying when hungry, excitement when presented with breast)	Eagerly opening mouth to feed Crying when hungry
Loss of interest on the breast quickly (short lived latch, lack of effort to suck, did not make eye contact with mother while feeding)	Interested in breastfeeding (content on the breast, effective suck to transfer milk for full feeding, gazed at mother while feeding)
Very particular about positioning while breastfeeding (needed movement while feeding, pushed off the breast often, very irritable when breast position changed)	Able to breastfeed in several positions, did not push off the breast, was able to breastfeed in several distinct locations outside the home
Uncoordinated latch / suck and difficulty coordinating swallowing with milk letdown	Coordinated latch with strong suck and no issues swallowing with strong milk letdown
Lack of regulation with feeding (constantly wanting to eat despite adequate milk intake)	Satiated after feedings and breastfed in age-appropriate intervals
Difficulty latching despite tongue tie revision	Siblings also with tongue tie but did not require revision and did not have latching difficulty
Choking and gagging while breastfeeding	Lack of gagging, choking, or coughing while breastfeeding
Poor milk transfer with weak suck despite adequate milk supply	Efficient suck with appropriate milk transfer
Lack of effort to latch and suck and was most effective eating only when asleep and relaxed	Strong suck and same effort / strength with suck when awake and asleep
Would fatigue easily on the breast and would fall asleep leading to persistent incomplete feeds	Latching difficulty the first two weeks but then resolved and breastfed without difficulty
Persistent painful latch for entire year of breastfeeding with no evidence of anatomic abnormality	No pain with latching

#### Mother’s Detection of Different Breastfeeding Behaviors in Neurotypical & Index Infant

When asked if they could identify different breastfeeding behaviors in their index infant as compared to their neurotypical infant, 18 mothers were able to recognize that the breastfeeding experience with their index infant was much different. Mothers reported that their index infant did not stay on the breast and would lose interest quicker than their neurotypical sibling, the index infant drank slower than the neurotypical sibling, neurotypical sibling was able to feed in several positions as compared to index infant, and neurotypical infant exhibited a more coordinated suck and a lack of gagging. One mother recalls the difference she noticed in breastfeeding her first born neurotypical child and her autistic second child:

It was just not ever a consistent latch or drink without any extra movements and then be done. It was a lot of, I think now that I know a little bit more, I think it was maybe some oral defensiveness, but just a lot of him kind of slightly pushing away the whole time that he was nursing, but he also loved it. He was very happy to nurse too, but you could just tell it was different for sure from my daughter.

#### Ease of Breastfeeding Neurotypical Sibling

Of the 24 mothers interviewed, 23 reported that breastfeeding their neurotypical child was much easier than breastfeeding their autistic child. Three mothers attributed this to maternal factors such as better milk supply and feeling more relaxed with breastfeeding in general with their neurotypical child due to birth position and prior experience. One mother remembers, “As I’m thinking about it with him (index infant), for some reason, my milk never came in. I remember with my daughter (neurotypical infant), waking up like soaked one day. Never ever happened with him.”

The remaining 20 mothers identified that because their neurotypical child did not possess the problematic feeding behaviors they had with their autistic child, breastfeeding was much easier. One mother describes the difference in breastfeeding her first born neurotypical child and second born autistic child who are both female as:

My first child had a really strong latch and I could nurse her like a football. I mean, I could nurse her in all sorts of ways. With my second, it had to be that specific way in position so she could actually get a strong latch there because it was a very soft suckle.

## Discussion

This study reports the breastfeeding behaviors that caused difficulty for mothers to breastfeed and the rationale behind breastfeeding cessation of infants later diagnosed with autism. It also highlights the different breastfeeding experiences mothers had with their autistic child and their neurotypical child. The challenges to breastfeeding their autistic child successfully included latching issues, maternal factors such as decreased milk supply and problematic anatomy, and maternal physical and mental health.

While these factors are commonly reported in the breastfeeding literature (Morrison et al., 2019), additional problematic breastfeeding behaviors by the index infant were identified through the maternal interviews that may indicate other contributors to breastfeeding difficulty including oral motor issues, sensory sensitivities, social issues, and regulation difficulties. An uncoordinated latch, choking, and gagging with breastfeeding were reported by six mothers, one of which was a healthcare professional who recognized by a week of age that her infant had low muscle tone. Hypotonia in infants can affect the muscles around the mouth and neck and can affect the abilities of the infant to suck and feed effectively (Bodensteiner, 2008). Gabis et al. (2021) found that hypotonia was a significant comorbidity in autistic children who were diagnosed at age 2.5 years and younger and may serve as a “red flag” for earlier identification of neurodevelopmental dysfunction and autism evaluation in a large cohort study. Poor suck, choking, and gagging with breastfeeding as described by the mothers in this study may indicate further assessment is needed for hypotonia or other neuromotor issues.

Social behaviors that impeded breastfeeding were reported by mothers that included their index infant losing interest in breastfeeding after a few minutes despite inadequate intake, difficulty focusing on breastfeeding even at a very young age, and a lack of hunger cues that they did not experience with their neurotypical child. Feeding involves social interaction and the hunger signals that infants typically display to communicate hunger in the first 5 months of life include waking up to eat, crying when hungry, and opening their mouth to imply wanting to feed more (Perez-Escamilla et al., 2017). Similar results were reported by Dewrang and Sandberg (2009) with parents reporting difficulty discerning hunger in their young autistic child. Further work is warranted to explore if these lack of hunger cues are early signs of the persistent deficits in social communication and social interaction that are part of the DSM-5 diagnostic criteria for autism (APA, 2022).

Mothers also reported that their autistic child was never satiated despite an adequate intake of milk. This report is similar to those of the mothers of autistic children in Lucas and Cutler’s (2015) study of a dysregulated feeding pattern of vigorous sucking in infants that may indicate an early sign of a persistent repetitive behavior, one of the criteria for autism diagnosis. The constant need to eat also brings speculation about an early lack of regulation in these infants in regards to their ability to identify when they are hungry or satiated. On the contrary, other infants were found to fall asleep quickly or lack the motivation or stamina to continue breastfeeding despite feeding inadequate amounts. In Lemcke et al.’s (2018) study, children at 18 months of age who were described as less active than their same age peers were found to have a higher risk of autism but these behaviors were not studied at a younger age.

Several mothers discussed the need for their autistic child to be breastfed in a certain position or they would refuse to eat, which was a different experience from breastfeeding their neurotypical child. Studies on older autistic children by Huxham et al. (2019) and Provost et al. (2010) found that they displayed significantly more ritualistic eating behaviors such as sitting in one particular chair for each meal or refusing to eat in other locations outside of the home compared to their neurotypical peers. Further investigation is needed to explore if preference of breastfeeding in one certain position is an early sign of the rigidity and routine behaviors that are displayed by autistic children.

Some mothers, when recalling their breastfeeding experiences, identified signs of sensory sensitivity in their index infants. Pushing back off the breast, screaming with latching, and never seeming content on the breast were all characteristics mothers looked back on and questioned whether it was due to sensory sensitivities as they were not present in neurotypical siblings. Sensory differences in autistic individuals is extremely common with tactile sensitivity being one of the most prominent elements affected (Panerai et al., 2023). Tactile defensiveness can be exhibited by autistic children in several ways, including pulling away when touched or difficulty with being cuddled (Cermak et al., 2010). Breastfeeding is highly tactile and puts into question whether these reported behaviors of backing off of the breast and never being content on the breast are early signs of tactile defensiveness.

These findings warrant further study of these breastfeeding behaviors to ascertain how common these behaviors are in infants later diagnosed with autism and if they can be used to help identify early signs of autism. If found to be prevalent in infants later diagnosed with autism, they may be helpful for primary care providers and lactation consultants to be aware of when assessing infants during routine visits. Additionally, in future studies focusing on breastfeeding duration in autistic children, asking participants about these breastfeeding behaviors may provide more context as to why there is contradicting evidence on shorter breastfeeding duration in infants ultimately diagnosed with autism (Emond et al., 2010; Husk & Keim, 2015; Lemcke, 2018; Soke et al., 2019).

It is important to note that five of the mothers reported feeding difficulty with their autistic child that persists today. One child has a formal diagnosis of Avoidant Restrictive Food Intake Disorder (ARFID), a feeding disturbance that is characterized by persistent inadequate intake to meet appropriate energy and/or nutritional needs (APA, 2022). Two other children meet the diagnostic criteria for Pediatric Feeding Disorder (PFD) as they still need food textures modified so that they can safely eat solid foods (Goday et al., 2019). The remaining two children struggle with picky eating. This calls into question if particular breastfeeding challenges are indicative of future feeding disorders or if they are more specific to later autism diagnosis. It is imperative that future studies that focus on problematic breastfeeding behaviors in autistic children take into account subsequent diagnosis of ARFID or PFD to further investigate if any breastfeeding behavior is more specific to a feeding disorder or to ASD.

There are several limitations to our study. The discussion of mothers’ challenges breastfeeding their autistic children took place in the context of an interview that had a broader range of feeding difficulties including breastfeeding. Because we were specifically focusing on feeding challenges in infancy, we acknowledge that our sample is not representative of all autistic children as not all autistic individuals experience feeding difficulties. Despite our efforts to recruit a diverse sample, the majority of our sample were White mothers whose autistic child was male. Although autism is more prevalent in White males, including more mothers of female autistic children and those of differing ethnic backgrounds would add additional breadth and context to this study. Lastly, this study used retrospective recall of the mothers’ breastfeeding experiences so there is a risk for recall bias. Several probes were included in the interview guide to help mothers remember certain events and mothers were able to reference materials such as baby books and medical records to aid them in recalling their infant’s feeding journey during the first year of life. Additionally, breastfeeding initiation has been found to be accurate up to 50 years later through maternal report although duration can be overestimated (Li et al., 2008).

## Conclusion

In summary, mothers reported several infant breastfeeding behaviors and maternal factors that contributed to difficulty breastfeeding and the cessation of breastfeeding in their autistic child. Mothers also described breastfeeding behaviors in their autistic child that were not present with their neurotypical child that may be indicative of early sensory sensitivity, lack of regulation, repetitive behaviors, and impaired social behaviors. These behaviors justify that further research is needed to discern if these breastfeeding behaviors can be used by primary care providers and lactation specialists to assess for neurodevelopmental concerns at a young age.
